# Supplementary material for: DMRforPairs: identifying Differentially Methylated Regions between unique samples using array based methylation profiles
Source: BMC Bioinformatics. 2014 May 15;15:141. doi: 10.1186/1471-2105-15-141 (PMC4046028; doi:10.1186/1471-2105-15-141)
Supplement: Additional file 2 — DMRforPairs output for the comparison of A431-MCF7 and NA17018-NA17105. Please start from the HTML files in each folder. Available via the BMC Bioinformatics website. [file 1471-2105-15-141-S2.zip › 1394847754114233_MOESM2_ESM/A431_MCF7/figures/17218.pdf]

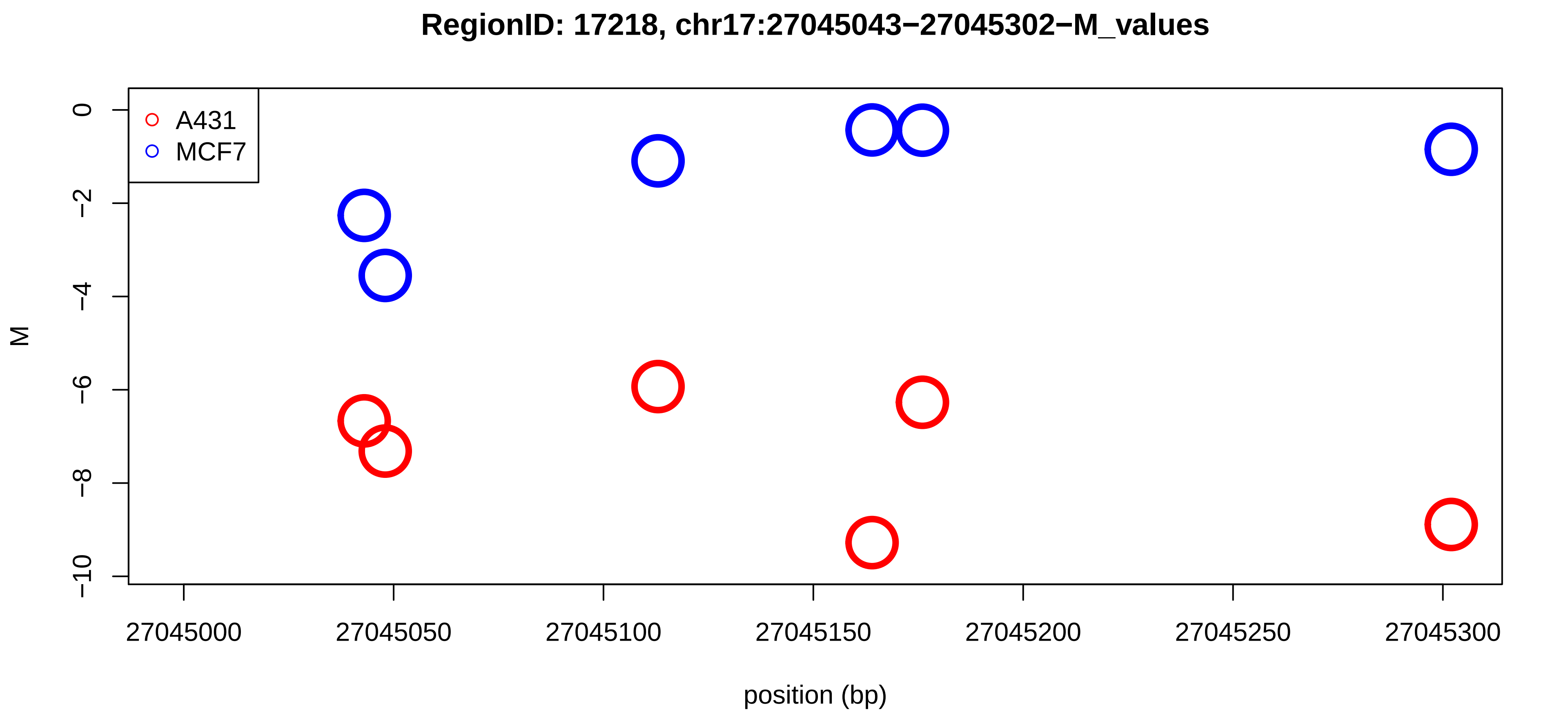

RegionID: 17218, chr17:27045043−27045302−M_values
A431
MCF7
M
0
−2
−4
−6
−8
−10
27045000
27045050
27045100
27045150
27045200
27045250
27045300
position (bp)

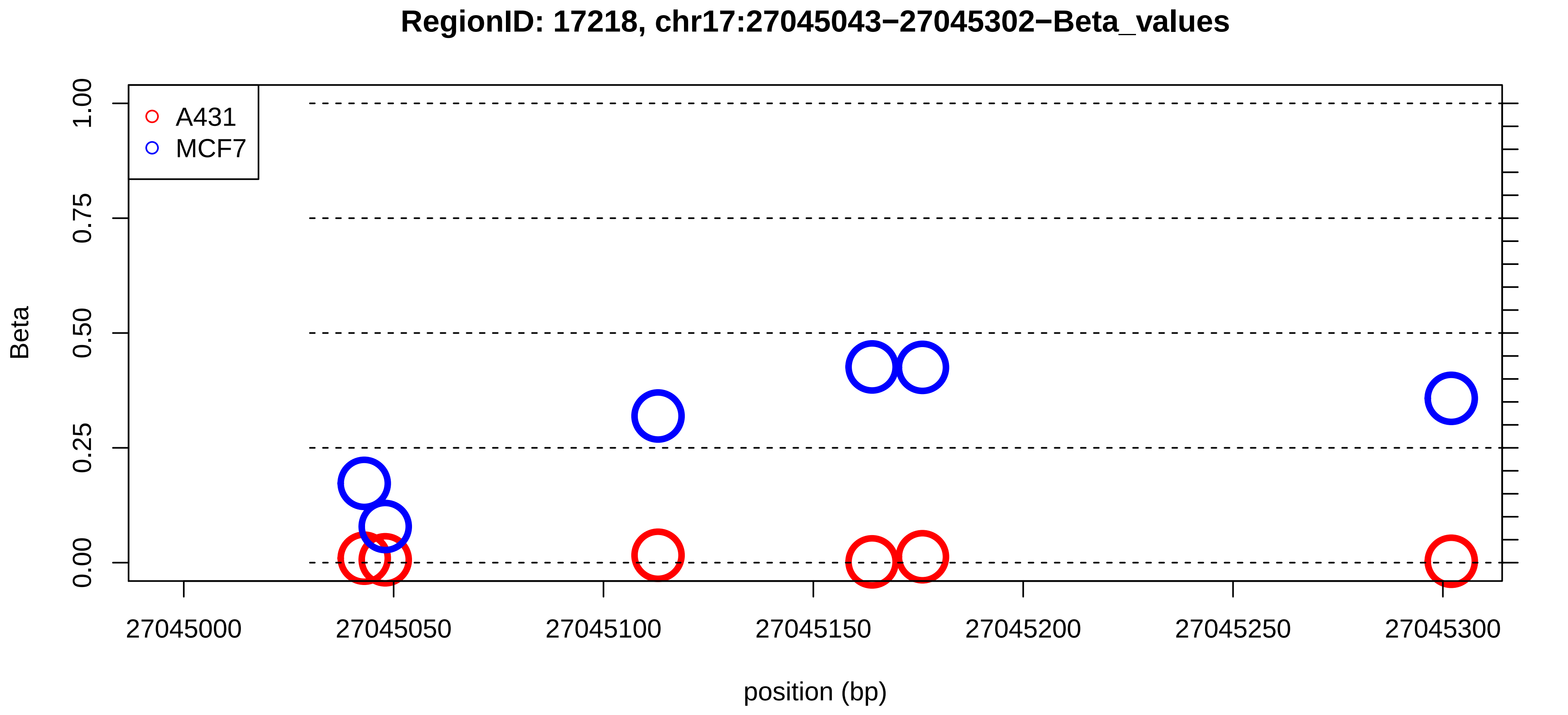

RegionID: 17218, chr17:27045043−27045302−Beta_values
A431
MCF7
Beta
1.00
0.75
0.50
0.25
0.00
27045000
27045050
27045100
27045150
27045200
27045250
27045300
position (bp)

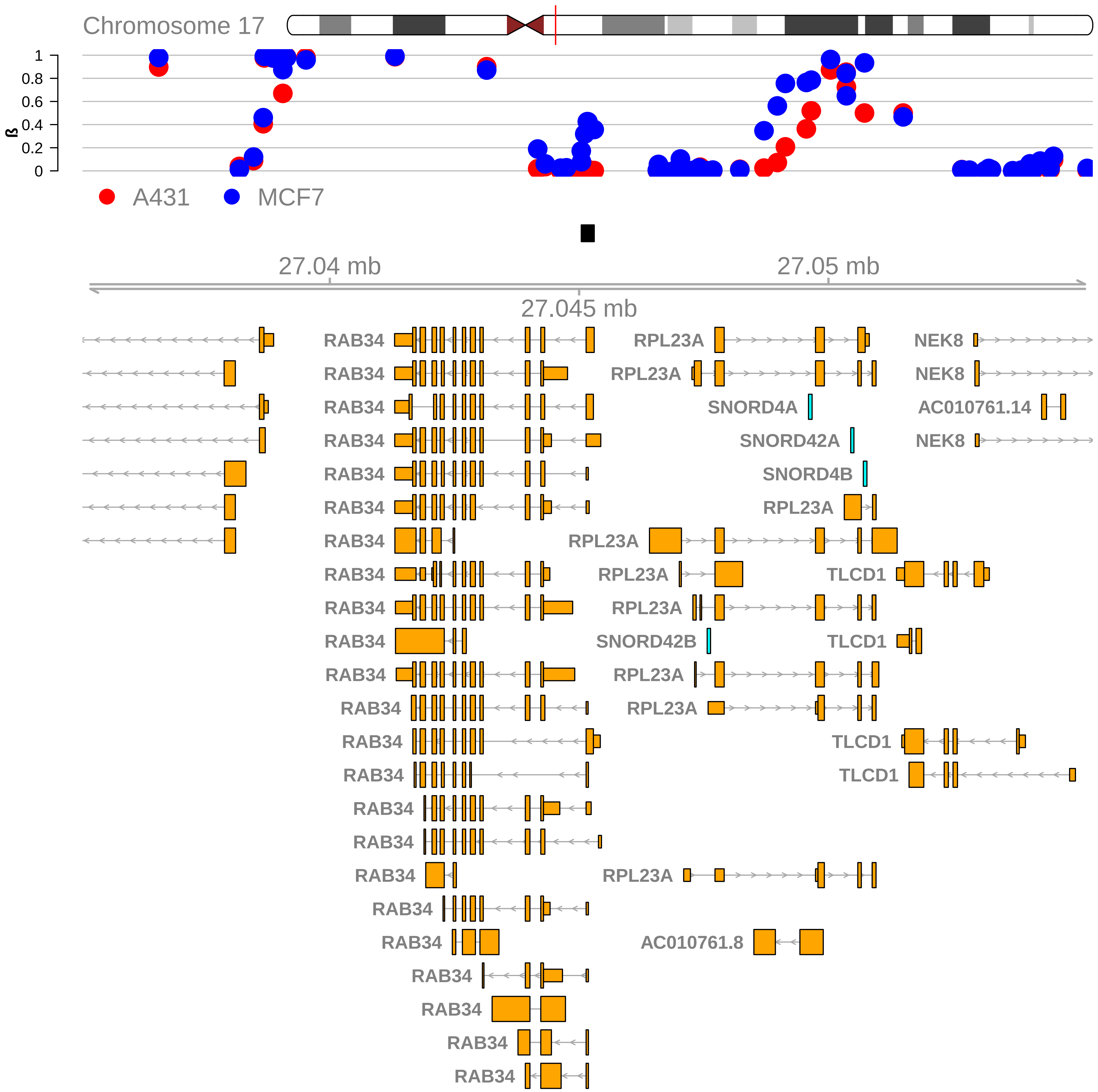

Chromosome 17
ß
1
0.8
0.6
0.4
0.2
0
A431
MCF7
27.04 mb
27.05 mb
27.045 mb
RAB34
RPL23A
NEK8
SNORD4A
AC010761.14
SNORD42A
SNORD4B
TLCD1
SNORD42B
AC010761.8
